# Insulin secretion impairment in Sirt6 knockout pancreatic β cells is mediated by suppression of the FoxO1-Pdx1-Glut2 pathway

**DOI:** 10.1038/srep30321

**Published:** 2016-07-26

**Authors:** Mi-Young Song, Jie Wang, Sun-O Ka, Eun Ju Bae, Byung-Hyun Park

**Affiliations:** 1 Department of Biochemistry, Chonbuk National University Medical School, Jeonju, Jeonbuk 54896, Republic of Korea; 2 College of Pharmacy, Woosuk University, Wanju, Jeonbuk 55338, Republic of Korea

## Abstract

Sirtuin 6 (Sirt6), a chromatin associated class III deacetylase, controls whole-body energy homeostasis and has a critical role in glucose-stimulated insulin secretion (GSIS) in pancreatic β cells. However, its underlying molecular mechanism remains poorly understood. To gain further insights, we studied the pathway by which Sirt6 regulates GSIS utilizing mice lacking Sirt6 in their β cells (βS6KO). Further, we overexpressed wild type or deacetylase-inactive mutant Sirt6 in isolated islets as well as in MIN6 cells. We confirmed that βS6KO mice developed glucose intolerance with severely impaired GSIS. Gene expression analysis of knockout islets and overexpression studies demonstrated that Sirt6 deacetylates forkhead box protein O1 (FoxO1) to trigger its nuclear export and releases its transcriptional repression of key glucose sensing genes such as *Pdx1* and *Glut2.* Ectopic overexpression of Sirt6 in knockout islets resulted in rescue of the defective insulin secretion and restoration of the expression of Pdx1 and Glut2. These results show that Sirt6 in pancreatic β cells deacetylates FoxO1 and subsequently increases the expression of Pdx1 and Glut2 to maintain the glucose-sensing ability of pancreatic β cells and systemic glucose tolerance.

Pancreatic β cells secrete insulin to maintain glucose homeostasis. In response to changes in circulating glucose concentration, glucose-sensing mechanisms in β cells are activated. The first-line mechanism is the glucose transporter Glut2. Because of its high K_m_ for glucose and high transport capacity, Glut2 allows for fast equilibration of the glucose concentration between the outside and inside of the cell^1^. In diabetes, reduced expression of *Slc2a2/Glut2* and impaired glucose-stimulated insulin secretion (GSIS) are observed^2^. Thus, glucose uptake through Glut2 is a key event for the control of GSIS in the diabetic state. The transcription of *Glut2* is regulated by pancreatic duodenal homeobox 1 (Pdx1)^3^. The ectopic expression of Pdx1 alone results in the induction of Glut2, whereas the dominant negative suppression of Pdx1 function or β cell-specific genetic deletion of *Pdx1* in mice drastically and selectively reduces the expression of Glut2, suggesting that Pdx1 is a master transcription factor in the regulation of Glut2^3^,^4^. Likewise, diabetic patients and mice have been shown to have mutation or decreased expression of Pdx1^5^,^6^.

The expression of Pdx1 is under the control of forkhead box protein O1 (FoxO1). Binding of FoxO1 to the *Pdx1* promoter negatively regulates transcription of this gene^7^. Transgenic overexpression of FoxO1 in the β cells results in decrease of *Pdx1* transcription and thus results in defective GSIS and impaired glucose tolerance in mice^8^. Conversely, haploinsufficiency of FoxO1 restores Pdx1 expression in β cells and rescues *insulin receptor substrate 2* knockout (KO) mice from developing diabetes^9^. In addition, FoxO1 and Pdx1 exhibit mutually exclusive patterns in subcellular localization. FoxO1 localizes in the cytosol of Pdx1-positive β cells, while it localizes in the nucleus of Pdx1-negative β cells^9^. Taken together, these results indicate that FoxO1 inhibits glucose sensing in β cells through direct suppression of Pdx1 expression and is critical for the maintenance of β cell function under stress conditions.

Post-translational modifications of FoxO1 influence its subcellular location and protein stability in response to various stimuli. Phosphorylation of FoxO1 causes the translocation of FoxO1 from the nucleus to the cytoplasm, where it is degraded via the ubiquitin-proteasome pathway^10^. Acetylation of FoxO1 also influences its DNA binding properties and its subcellular location, as hyperacetylation of FoxO1 shifts its equilibrium from a predominant cytosolic location toward nuclear accumulation^11^,^12^,^13^. *In vitro* experiments have demonstrated that ubiquitin-dependent degradation is accelerated in 6KR mutants in which six lysine residues corresponding to proposed FoxO1 acetylation sites were substituted with arginine (K242R, K245R, K259R, K262R, K271R, and K291R)^14^.

Sirtuin, a class III deacetylase, affects the lifespan of lower eukaryotes by deacetylating the *C. elegans* FoxO ortholog *daf-16*^15^. Among the seven members of the sirtuin family in mammals, Sirt1 has been shown to deacetylate FoxO1 and regulates the transcription of genes involved in diverse cellular processes such as proliferation^16^, gluconeogenesis^17^, adipogenesis^18^, and osteoblastogenesis^19^. In terms of FoxO1 deacetylation, much less is known about the function of the other sirtuins. Sirt6, a chromatin-associated deacetylase, preferentially deacetylates histone H3 lysine 9 (H3K9) and histone H3 lysine 56 (H3K56) and acts as a repressor of nuclear factor κB, c-JUN, and hypoxia-inducible factor 1α^20^,^21^. It also deacetylates non-histone proteins such as FoxO1^22^, C-terminal binding protein interacting protein^23^, and acetyltransferase GCN5^24^. In this study, we investigated whether Sirt6 could regulate the glucose-sensing mechanism and concomitant insulin secretion from β cells. To address this issue, we generated β cell-specific *Sirt6* KO mice (βS6KO) and analyzed their metabolic phenotypes. To provide mechanistic interpretation, we overexpressed wild type (WT) or deacetylase-inactive Sirt6 in MIN6 cells as well as in isolated islets and assessed the FoxO1-Pdx1-Glut2 pathway.

## Results

### Sirt6 protein levels in pancreatic islets are decreased under diabetic conditions

To investigate potential changes in Sirt6 in pancreatic islets under diabetic conditions, we first analyzed Sirt6 expression levels in various pathologic conditions linked to diabetes. The Sirt6 protein levels in the mouse islets were markedly decreased by incubation of either cytokine mixtures or palmitate (Fig. S1A). Similarly, islets isolated from high fat diet (HFD)-fed mice and pancreatic tissues from streptozotocin-treated mice and *db/db* mice showed lower expression levels of Sirt6 compared with their control groups (Fig. S1A,B). To identify the specific cell types of the pancreas in which Sirt6 was expressed, mice pancreas sections were stained with antibodies specific for Sirt6, insulin, or glucagon. Results indicated that Sirt6 co-localized mainly with insulin and to a lesser extent with glucagon (Fig. S1B). A decrease in β cell mass in streptozotocin-treated and *db/db*-mice was confirmed by H&E and insulin immunostaining.

### Sirt6 deletion in pancreatic β cells causes glucose intolerance in mice and defective insulin secretion in β cells

To investigate the physiological role of Sirt6 in pancreatic β cell function, we generated β cell-specific *Sirt6* KO mice (*Sirt6*^*fl/fl*^*:Rip2-Cre*) (Fig. S2A). Western blotting and immunohistochemistry results confirmed β cell-specific deletion of *Sirt6* in the islets of βS6KO mice (Fig. S2B,C).

**Figure 1:**
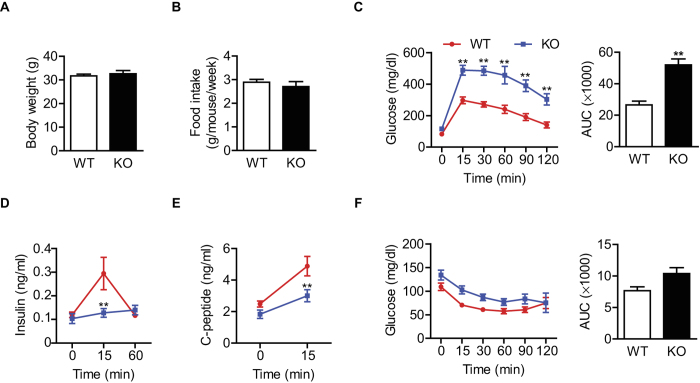
Effect of β cell-specific Sirt6 deletion on glucose homeostasis. (**A,B)** Body weight at 12 weeks of age and weekly food intake. **(C)** Plasma glucose concentrations during intraperitoneal glucose tolerance tests in 14-h fasted mice. Areas under the curve were compared. **(D,E)** Glucose (1 g/kg) was administrated intraperitoneally to 14-h fasted mice. Plasma insulin and C-peptide levels were measured at indicated time points. **(F)** Plasma glucose concentrations during insulin tolerance tests in overnight fasted mice. Areas under the curve were compared. Values are mean ± SEM (n = 5–7). ^**^p < 0.01 vs. WT.

βS6KO and WT littermate mice had similar weight gain and food intake during the first 12 weeks of life (Fig. 1A,B). We performed the glucose tolerance test (GTT) in 12-week-old mice. Under basal conditions, blood glucose and insulin levels did not differ between βS6KO and WT mice (Fig. 1C,D). However, glucose tolerance was significantly impaired in βS6KO mice, with a two-fold increase in AUC compared to WT mice (Fig. 1C). The remarkable glucose intolerance that developed in βS6KO mice was accompanied by defective insulin secretion, which was observed as significant reduction in insulin and C-peptide levels after glucose load (Fig. 1D,E). The results of the insulin tolerance test (ITT) were not significantly different between WT and βS6KO mice (Fig. 1F). These phenotypes were recapitulated in younger βS6KO mice (i.e., by 4 week) compared to age-matched WT mice (Fig. S3). In order to investigate whether the defect in insulin secretion upon glucose stimulation in βS6KO mice resulted from an impairment in islet development or insulin production, we performed an immunostaining assay for insulin in the islets. The results did not reveal any differences in the insulin-positive area, cellular arrangements, or compositions between the WT and βS6KO mice, with the majority of β cells staining positive for insulin and with glucagon-positive α-cells in the periphery region (Fig. S4A–C). Quantitative analyses of the pancreatic insulin contents (Fig. S4D) and plasma glucagon levels (Fig. S4E) confirmed the histological results. Because an apparent dysfunction of β cells can be caused by enhanced apoptosis, we performed TUNEL staining in pancreatic tissues (data not shown) but did not observe any difference in the apoptotic index between genotypes (Fig. S4F). These results suggest that the Sirt6 deletion in β cells does not affect islet development, apoptosis, or insulin content. Rather, insulin secretion defects underlie the glucose intolerance of βS6KO mice.

Next, to determine whether a change in the calcium flux of β cells, which is the last step of GSIS, is involved in Sirt6 deletion-mediated defects in insulin secretion, we examined the direct effects of WT or mutant Sirt6 overexpression on the intracellular Ca^2+^ concentration ([Ca^2+^]_i_). When stimulated with 20 mM glucose, MIN6 cells infected with AdSirt6 but not with a catalytically inactive mutant *Sirt6-H133Y* (AdmSirt6) showed greater [Ca^2+^]_i_ oscillations compared to the AdLacZ-infected cells, although at 2 mM glucose, the [Ca^2+^]_i_ did not differ among groups (Fig. S5A,B), suggesting that Sirt6 with intact deacetylase activity is required for the regulation of calcium flux in β cells. The GSIS results of a study conducted in MIN6 cells infected with AdSirt6 demonstrated an intimate correlation between [Ca^2+^]_i_ and insulin secretion (Fig. S5C). Further GSIS studies were performed in the islets obtained from WT and βS6KO mice. Confirming the defect in GSIS in βS6KO mice and the ectopic Sirt6-mediated GSIS increase in MIN6 cells, marked impairment of GSIS was observed in the islets from βS6KO mice (Fig. S5D). Together, these data indicate that Sirt6 is a critical mediator of calcium flux and insulin secretion in response to glucose stimulation that is necessary for the maintenance of glucose tolerance.

### Sirt6 deacetylates FoxO1 and regulates the FoxO1-Pdx1-Glut2 pathway in β cells

**Figure 2:**
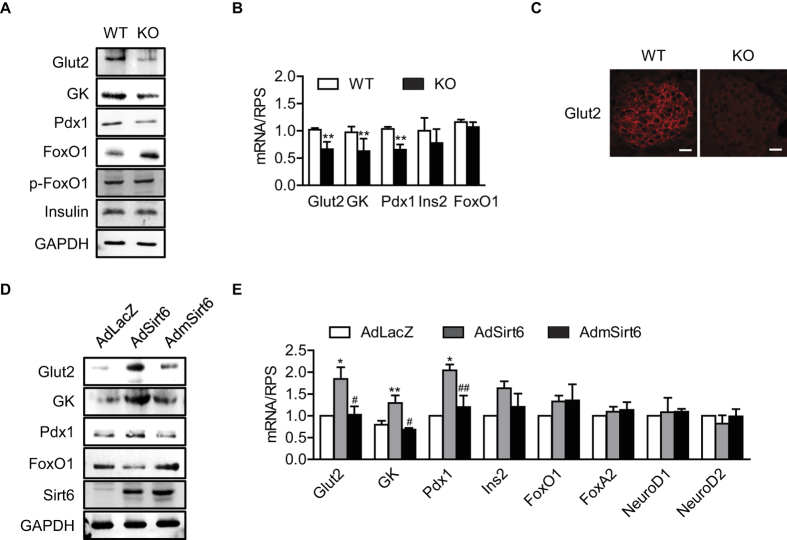
Regulation of FoxO1-Pdx1-Glut2 pathway by Sirt6. **(A,B)** Islets were isolated from WT and βS6KO mice, and protein and mRNA levels of indicated genes were analyzed by Western blotting or real-time RT-PCR, respectively. **(C)** Immunofluorescence staining of Glut2 in pancreatic islets. Bars = 20 μm. **(D,E)** Islets were isolated from c57BL6 mice and infected with control or Sirt6 adenoviruses, and protein and mRNA levels of indicated genes were analyzed. Values are mean ± SEM (n = 6). ^*^p < 0.05 and ^**^p < 0.01 vs. WT or AdLacZ; ^#^*p* < 0.05 and ^##^*p* < 0.01 vs. AdSirt6.

GSIS in β cells is driven by glycolysis and glucose transport through Glut2, which is the first step in the glucose-sensing mechanism^1^. Given that Sirt6 is known to directly deacetylate FoxO1^22^, which binds to the *Pdx1* promoter and downregulates its transcription^3^, and that Pdx1 upregulates *Glut2* transcription^7^, we predicted that Sirt6 regulates GSIS by interacting with FoxO1. To test this hypothesis, we compared the expression levels of Glut2, glucokinase (GK), Pdx1, and FoxO1 in islets from WT and βS6KO mice. We observed a significant decrease in the expression of Glut2, GK, and Pdx1 in βS6KO islets in terms of both protein and mRNA levels (Fig. 2A,B). On the contrary, the FoxO1 protein level was increased in βS6KO islets without changes in either the mRNA level or its phosphorylation status. The immunofluorescence staining result confirmed the Glut2 decrease in the βS6KO islets (Fig. 2C). In the converse approach, the infection of islets with AdSirt6 increased mRNA and protein levels of Glut2, GK, and Pdx1 and decreased FoxO1 protein expression (Fig. 2D,E), indicating the presence of a causal relationship between Sirt6 and FoxO1-Pdx1-Glut2. Moreover, AdmSirt6 infection had no effect, confirming the role of the deacetylase-specific activity of Sirt6 on the regulation of the FoxO1-Pdx1-Glut2 pathway.

**Figure 3:**
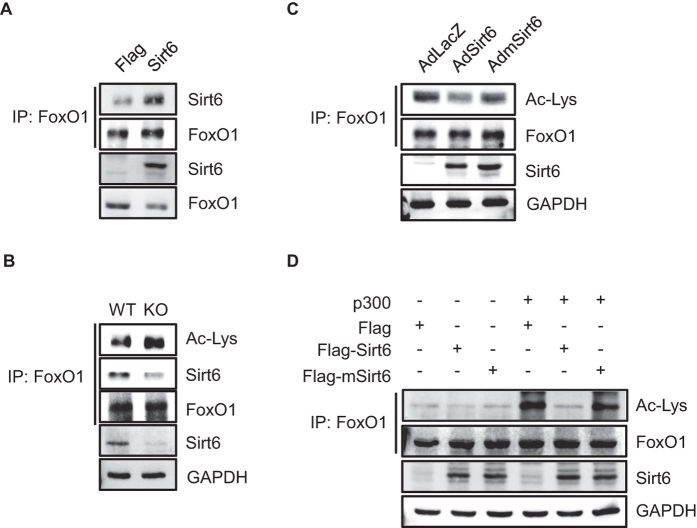
Deacetylation of FoxO1 by Sirt6. **(A)** HEK293 cells were transfected with *Flag* or *Sirt6*, and interaction between Sirt6 and FoxO1 was analyzed. **(B)** Islets were isolated from WT and βS6KO mice, and acetylation of FoxO1 was compared. **(C)** Islets isolated from c57BL6 mice were infected with control or Sirt6 adenoviruses, and then acetylation of FoxO1 was compared. **(D)** HEK293 cells were transfected with plasmids as indicated, and deacetylation of FoxO1 was analyzed.

Because there was no difference in p-FoxO1 levels between the genotypes (Fig. 2A) and Sirt6 had a deacetylase-dependent effect on GSIS (Fig. S5C), we examined FoxO1 acetylation in addition to the possible interaction of FoxO1 and Sirt6. The results of co-immunoprecipitation assays demonstrated a direct interaction between FoxO1 and Sirt6 in HEK293 cells overexpressing Sirt6 (Fig. 3A). When FoxO1 immunoprecipitates obtained from islet lysates were immunoblotted with anti-acetyl lysine (Ac-Lys) antibody, the level of FoxO1 acetylation was increased in βS6KO mice (Fig. 3B) and decreased by Sirt6 overexpression (Fig. 3C), indicating the role of Sirt6 in the control of FoxO1 acetylation. To further validate this result, HEK293 cells were co-expressed with acetyltransferase p300 along with Sirt6 or mSirt6. As expected, FoxO1 acetylation was increased by p300 overexpression but decreased by Sirt6, with no effect by mSirt6, confirming that FoxO1 is a direct deacetylation target of Sirt6 (Fig. 3D).

**Figure 4:**
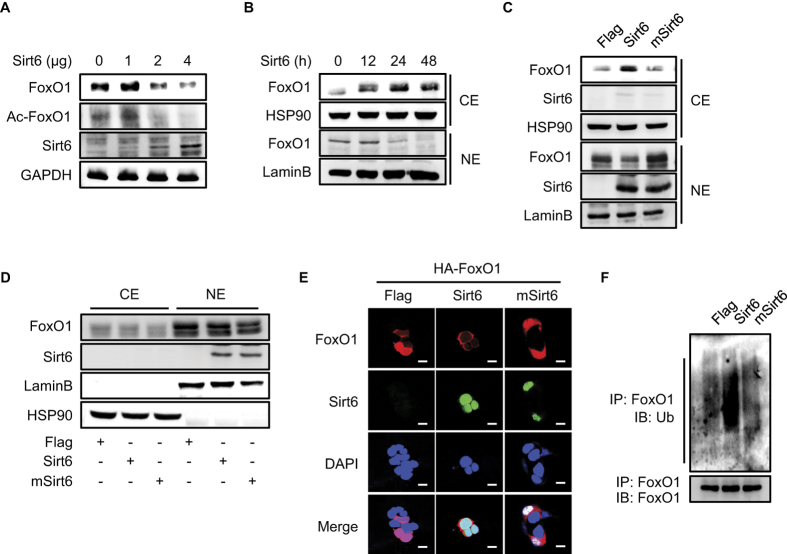
Nuclear-to-cytosolic trafficking and proteasome-dependent degradation of FoxO1 by Sirt6. **(A)** HEK293 cells were transfected with indicated amounts of *Sirt6*, and the levels of total- and acetylated-FoxO1 were analyzed. **(B)** HEK293 cells expressing Sirt6 were treated with 2 μM of MG132 for indicated time periods, and protein levels of FoxO1 in cytosolic extract (CE) and nuclear extract (NE) were determined. **(C,D)** HEK293 cells expressing Sirt6 or mSirt6 were treated with either 2 μM MG132 for 24 h (**C**) or 0.5 ng/ml leptomycin B for 12 h (**D**), and FoxO1 levels in cytosolic and nuclear extracts were analyzed. **(E)** After transfection with the indicated plasmids, HEK293 cells were subjected to immunofluorescence staining. **(F)** Forty-eight hour post-transfection in the presence of MG132, total cell lysates were immunoprecipitated with anti-FoxO1-antibodies and immunoblotted with anti-ubiquitin antibodies.

To determine the consequence of Sirt6-mediated FoxO1 deacetylation, we next examined the nuclear/cytoplasmic shuttling and protein degradation of FoxO1. In HEK293 cells transfected with increasing amounts of *Sirt6*, we found that exogenous Sirt6 dose-dependently decreased the total level of FoxO1 protein, as well as its acetylation (Fig. 4A). To test whether the decrease in FoxO1 protein level by Sirt6 was due to protein degradation in the cytosol, we repeated the experiments in the presence of MG132, a proteasome inhibitor. As a function of post-transfection time of Sirt6, nuclear FoxO1 level was decreased, while its cytosolic level was increased (Fig. 4B), with no effect on mutant *Sirt6*-transfected cells (Fig. 4C), indicating that deacetylase activity is required for the Sirt6-dependent nuclear export of FoxO1. Many nuclear-to-cytoplasmic translocating proteins depend on the nuclear export receptor Crm-1 for nuclear exit^25^. Treatment of cells with leptomycin B, an inhibitor of Crm-1, completely prevented the Sirt6-mediated FoxO1 translocation to the cytosol (Fig. 4D), indicating the presence of Crm-1-dependent FoxO1 nuclear exit by Sirt6. Confocal microscopic observation further confirmed that a complete shift of FoxO1 from the nucleus to the cytosol occurred in the presence of Sirt6 but not in the presence of mSirt6, whereas FoxO1 was predominantly localized in the nucleus in the basal state (Fig. 4E). Once FoxO1 exits to the cytosol, it quickly degrades in the proteasome following ubiquitination. Accordingly, the ectopic expression of Sirt6 significantly increased FoxO1 ubiquitination (Fig. 4F), supporting the notion that Sirt6 causes the deacetylation, cytosolic translocation, and proteasomal degradation of FoxO1. When the experiments were repeated in MIN6 cells, we obtained identical results as in HEK293 cells (Fig. S6).

**Figure 5:**
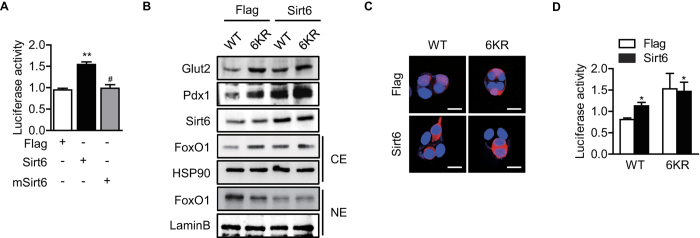
Regulation of Pdx1 and Glut2 expression by acetylation-deficient FoxO1 (6KR) mutant. **(A)** MIN6 cells were transiently transfected with *Pdx1* luciferase construct together with *Flag or Sirt6*. After 48 h, cells were harvested in the reporter lysis buffer, and luciferase activity in cell lysates was assayed. **(B)** MIN6 cells were co-transfected with *Flag* or *Sirt6* along with either WT *FoxO1* or 6KR mutant *FoxO1* and treated with 2 μM of MG132 for 24 h. Protein levels of Glut2 in whole cell lysates and Pdx1 and FoxO1 in cytosolic (CE) and nuclear extract (NE) were determined. **(C,D)** After transfection as indicated, confocal microscopic analysis for subcellular localization of FoxO1 (**C**) and Pdx1 luciferase assay (**D**) were performed. Values are mean ± SEM (n = 6). ^*^p < 0.05 and ^**^p < 0.01 vs. Flag or Flag + WT FoxO1 FoxO1.

We investigated whether the Sirt6-induced nuclear export of FoxO1 led to a loss of inhibition of *Pdx1* transcription by transfection of MIN6 cells with the luciferase reporter construct containing the *Pdx1* promoter together with *FoxO1* and *Sirt6* cDNA. As shown in Fig. 5A, wild type Sirt6 significantly increased *Pdx1* luciferase activity, while mSirt6 had no effect. Next, to determine whether FoxO1 deacetylation by Sirt6 is essential for its nuclear export and the expression of Pdx1 and Glut2, we used a 6KR FoxO1 mutant which is a constitutively deacetylated form of FoxO1^14^. On Western blotting, the transfection of MIN6 cells with 6KR mutant increased protein levels of Pdx1 and Glut2 regardless of *Sirt6* transfection (Fig. 5B). Moreover, cytosolic FoxO1 level (Fig. 5B,C) and Pdx1 luciferase activity (Fig. 5D) were higher in 6KR *FoxO1*-transfected cells as compared to WT *FoxO1*-transfected cells. These results indicate that deacetylation of FoxO1 by Sirt6 resulted in the nuclear exit of FoxO1, which releases its transcriptional suppression of *Pdx1* and *Glut2*. To prove our hypothesis that deacetylation of FoxO1 may accelerate its protein degradation, we measured FoxO1 turnover by incubating MIN6 cells with cycloheximide after transfection of WT or 6KR *FoxO1* along with either control vector (*Flag*) or *Sirt6*. Cycloheximide treatment caused a decline in FoxO1 protein levels and FoxO1 degradation was further augmented by 6KR *FoxO1* transfection (Fig. S7). Furthermore, the protein stability of FoxO1 was also decreased in cells transfected with *Sirt6* compared to the cells with *Flag*, which may reflect the increased ubiquitination of FoxO1 by Sirt6 overexpression as shown in Fig. 4F. By double transfection of *Sirt6* and 6KR *FoxO1*, however, in contrast to our expectation, FoxO1 showed a reduced stability compared with Flag+6KR FoxO1 group and this suggests that there may be an additional mechanism regulating FoxO1 stability independently of the lysine deacetylation.

Pdx1 deficiency is closely related to mitochondrial dysfunction and defective insulin secretion through suppression of mitochondrial transcription factor A (TFAM)^26^. We therefore examined expression level of TFAM and noted it to be decreased in islets of βS6KO mice compared to islets of WT mice (Fig. S8A). Conversely, Sirt6 overexpression increased TFAM protein level, while mutant Sirt6 overexpression had no effect on its expression (Fig. S8B). Parallel to TFAM expression, ATP level after glucose stimulation was significantly decreased in islets of βS6KO mice (Fig. S8C).

### Overexpression of Sirt6 enhances GSIS in islets from βS6KO mice

**Figure 6:**
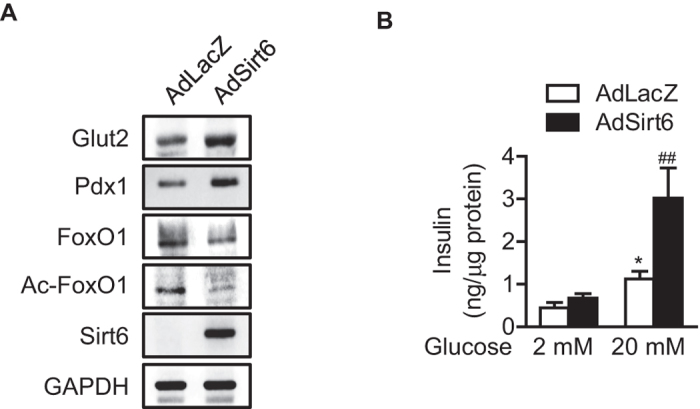
Effects of forced expression of Sirt6 on Pdx-1 and Glut2 protein levels and GSIS in βS6KO islets. **(A)** Islets were isolated from βS6KO mice and infected with AdLacZ or AdSirt6. Protein levels were determined in whole cell lysates. **(B)** After infection with AdLacZ or AdSirt6, GSIS was performed. Values are mean ± SEM (n = 6). ^*^p < 0.05 vs. 2 mM glucose; ^##^*p* < 0.01 vs. AdLacZ+20 mM glucose.

To confirm the important role of Sirt6 in insulin secretion, we finally tested whether Sirt6 gene transfer could rescue the functional defect seen in the islets of βS6KO mice. Ectopic expression of Sirt6 in βS6KO islets resulted in restoration of the protein levels of Pdx1 and Glut2 and reduction of acetylated- and total-FoxO1 (Fig. 6A). Accordingly, insulin secretion in response to 20 mM glucose was also fully restored by the forced expression of Sirt6 in the islets of βS6KO mice (Fig. 6B).

## Discussion

This study demonstrates that Sirt6 interacts with and deacetylates FoxO1 in β cells, which leads to the nuclear exclusion of FoxO1, alleviation of its inhibition of Pdx1 transcription, and the consequent upregulation of Glut2 that mediates insulin secretion upon glucose challenge. The plausibility of our initial hypothesis was derived from the following previous reports: (1) Sirt1 deacetylates FoxO1 and downregulates its transcriptional activity^16^,^17^,^18^,^19^; (2) Sirt6 regulates hepatic gluconeogenesis by promoting FoxO1 nuclear exclusion^22^, and (3) FoxO1 negatively regulates glucose sensing by suppressing the expression of *Pdx1*^8^. As such, this study points to the essential role of Sirt6 as a regulator of glucose sensing in β cells.

Studies have demonstrated that Sirt1 enhances the transcription and secretion of insulin, the latter of which occurs though the promotion of membrane depolarization and Ca^2+^ influx by repressing uncoupling protein 2 expression^27^. However, little is known about the role of the other sirtuins in insulin secretion. While we were preparing this manuscript, Xiong *et al*.^28^ reported that Sirt6 regulates GSIS through mitochondrial glucose oxidation, plasma membrane depolarization, and calcium dynamics. Although these results reveal a novel role of Sirt6 in the regulation of GSIS, the underlying molecular mechanism is still largely elusive. Starting from our initial observations and hypothesis, which were in agreement with the results of Xiong *et al*., we demonstrate that Sirt6 deletion in β cells leads to the inhibition of GSIS, which is mediated by the hyperacetylation and overactivation of FoxO1.

In this study, we provided evidence that Sirt6 regulates the key molecules for glucose sensing in β cells. First, the Sirt6 protein levels in the islets were decreased in *in vitro* and *in vivo* diabetic conditions, suggesting that Sirt6 is a regulator of β cell homeostasis under pathophysiological conditions. Second, β cell-specific Sirt6 deletion impaired glucose tolerance. Third, islets from βS6KO mice demonstrated reduced calcium influx and insulin secretion in response to 20 mM glucose, while Sirt6 overexpression stimulated insulin secretion. Analysis of gene expression in the islets revealed that *Glut2, GK*, and *Pdx1* were selectively downregulated in βS6KO mice, whereas the levels of *Ins2, NeuroD1, NeuroD2*, and *FoxO1* were unchanged, indicating that GSIS impairment does not result from alterations of insulin expression or β cell proliferation. Fourth, Sirt6 deletion, unlike its impact on GSIS, did not affect islets development. The cellular architecture and area of βS6KO islets were not different from the control islets, suggesting that Sirt6 expression is not required for normal β cell mass expansion during development.

Physiologically, Glut2, along with GK, has been implicated in playing a central role in the control of GSIS. Compared to GK, Glut2 functions in a permissive way by facilitating the entry of glucose and glycolysis at physiological glucose concentrations. Emerging evidence demonstrates that decreased level of Glut2 causes reduction in insulin secretion^29^,^30^, suggesting a prominent role of Glut2 in GSIS. On the other hand, others have suggested that GSIS can proceed normally even in the presence of low level of Glut2^31^. Therefore, the moderate reduction of Glut2 level observed in the islets of βS6KO mice may not be enough to explain the impaired glucose tolerance. The combined dysfunction of some other molecules such as GK and through mitochondrial dysfunction might have contributed to the significant impairment of insulin secretion.

What is the significance of Sirt6-mediated FoxO1 deacetylation in β cell physiology? FoxO1 activity, as measured by the degree of suppression of *Pdx1* reporter activity, was increased in *Sirt6*-transfected MIN6 cells. It is likely that Sirt6-mediated deacetylation of FoxO1 provoked nuclear extrusion of FoxO1, which relieved a molecular blockage on *Pdx1* expression. Results from the 6KR FoxO1 mutant support this concept. In addition, the deacetylation and subsequent nuclear export of FoxO1 were critical for FoxO1 protein stability, as shown by the findings that the forced expression of Sirt6 enhanced proteasomal degradation of FoxO1, while deacetylase-defective mutant Sirt6 decreased the polyubiquitination of FoxO1. This model is consistent with a recent study performed by Zhang *et al*.^22^, in which Sirt6 cooperates with p53 to deacetylate FoxO1, transport FoxO1 from the nucleus to the cytosol, and suppress the expression of gluconeogenic genes.

In summary, we confirmed that Sirt6 in β cells is required for the regulation of insulin secretion and glucose homeostasis. Importantly, we demonstrated that Sirt6-mediated FoxO1 deacetylation leads to its nuclear export and the restoration of Pdx1 expression, which in turn augments Glut2 expression and GSIS. Therefore, pharmacological activators of Sirt6 may be a promising therapeutic tool for promoting insulin secretion in patients with diabetes.

## Materials and Methods

### Cell culture

MIN6 cells (donated by Professor Shong M, Chungnam National University, Daejeon, Republic of Korea) were cultured in DMEM supplemented with 100 U/ml penicillin, 100 μg/ml streptomycin, and 15% fetal bovine serum. For calcium imaging, cells were grown on a collagen-coated confocal dish and incubated in Krebs-Ringer bicarbonate buffer [136 mM NaCl, 5 mM KCl, 1 mM NaH_2_PO_4_, 1 mM MgSO_4_, 5 mM NaHCO_3_, 1.0 mM CaCl_2_, and 20 mM HEPES (pH 7.4)] for 3 h before measurements.

### Transient transfections and reporter gene assays

Transient transfections were performed using Lipofectamine 2000 (Invitrogen, Carlsbad, CA, USA) according to the manufacturer’s instructions. Briefly, ∼80% confluent MIN6 or HEK293 cells were transfected with 3 μg of *Flag, Flag-Sirt6, Flag-mSirt6*, WT and 6KR mutant *FoxO1*, and *p300*. For the reporter gene assay, 3 μg of *Flag, Flag-Sirt6*, or *Flag-mSirt6* with 3 μg of *Pdx1* PH2 promoter luciferase were used. After 48 h, cells were harvested in reporter lysis buffer (Promega, Madison, WI, USA). Luciferase activity was determined in whole cell lysates using the Promega luciferase assay kit and expressed as relative light units.

### Calcium imaging

MIN6 cells on a collagen-coated confocal dish were incubated with 5 μM Fluo-4 AM (Invitrogen) in KRB buffer containing 1% BSA at 37 °C for 40 min. Changes in intracellular Ca^2+^ concentration ([Ca^2+^]_i_) were determined at 488 nm excitation/530 nm emission by an air-cooled argon laser system. The emitted fluorescence at 530 nm was collected using a photomultiplier. The image was scanned using a confocal microscope (Nikon, Tokyo, Japan). For the calculation of [Ca^2+^]_i_, the method of Tsien *et al*.^32^ was used with the following equation: [Ca^2+^]_i_ = K_d_(F − F_min_)/(F_max _− F), where K_d_ is 345 nM for Fluo-4, and F is the observed fluorescence level. Each tracing was calibrated for the maximal (F_max_) by the addition of ionomycin (8 μM) and for the minimal intensity (F_min_) by the addition of 50 mM EGTA at the completion of each measurement.

### Preparation of recombinant adenovirus

Adenoviruses expressing the *Sirt6* (AdSirt6), a catalytically inactive mutant *Sirt6-H133Y* (AdmSirt6), and *β-galactosidase* (AdLacZ) were prepared as described previously^33^.

### Animals

*Sirt6*^*flox/flox*^ mice with exons 2 and 3 of *Sirt6* flanked by *loxP* sites (B6;129-*Sirt6*^tm1Ygu^/J) and *RIP2-Cre* mice that expressed the *Cre* recombinase gene under the control of the *rat insulin 2* gene promoter (B6.Cg-Tg(*Ins2-Cre*)25Mgn/J) were obtained from The Jackson Laboratory (Bar Harbor, ME, USA). We generated β cell-specific *Sirt6* KO (βS6KO) mice by breeding *Sirt6*^*flox/flox*^ mice with *RIP2-Cre* mice. To avoid potential variations stemming from gender and genetic background, male mice from the F6 generation, *Sirt6*^*flox/flox*^; *RIP2-Cre* (βS6KO), and *RIP2-Cre* (WT), were used for the studies. Male mice older than 4 weeks of age were fed *ad libitum* either standard laboratory chow diet (New Brunswick, NJ, USA) for 12 weeks. Intraperitoneal glucose tolerance tests (1 g/kg of body weight) and insulin tolerance tests (0.75 U/kg of body weight) were performed after 14 h of fasting. All animal experiments were performed in accordance with the Guide for the Care and Use of Laboratory Animals published by the US National Institutes of Health (NIH Publication No. 85-23, revised 2011). The study protocol was approved by the Institutional Animal Care and Use Committee of Chonbuk National University (permit number: CBNU 2015-087).

### Biochemical analysis

Blood samples were collected after overnight fasting. Insulin, C-peptide, and glucagon were measured using specific ELISA kits (ALPCO, Salem, NH, USA).

### Islet isolation, ATP measurement, and GSIS assay

Islets were isolated from WT and βS6KO mice using the collagenase digestion method^34^. Islets were washed three times in Krebs-Ringer bicarbonate buffer (115 mM NaCl, 5 mM KCl, 24 mM NaHCO_3_, 1 mM MgCl_2_, 2.5 mM CaCl_2_, 25 mM HEPES, 0.1% bovine serum albumin, pH 7.4) containing 2 mM D-glucose. Insulin secretion assays were performed with 20 mM D-glucose. ATP content in the islets after glucose stimulation was measured using bioluminescent assay kit (Sigma-Aldrich, St Louis, MO, USA) following the manufacturer’s instructions.

### Immunohistochemistry and immunocytochemistry

Tissues were fixed (10% formalin solution in 0.1 M PBS), paraffin embedded, and cut into 4-μm sections on a microtome. Tissue sections were stained with hematoxylin and eosin (H&E) under standard conditions. MIN6 cells were fixed in 2% paraformaldehyde for 40 min. Both the sections and cells were permeabilized in 0.1% Triton X-100 in PBS and blocked in 5% normal goat serum and 0.1% Triton X-100 in PBS prior to incubation with primary antibody. Islets were stained using Sirt6 Ab (Cell Signaling Technology, Beverly, MA, USA), Glut2 Ab (Santa Cruz Biotechnology, Dallas, TX, USA), glucagon Ab (Bioworld Technology, St Louis Park, MN, USA), and insulin Alexa Fluor® 488 (eBioscience, San Diego, CA, USA) together with DAPI nuclear stain (Invitrogen). Images were acquired using an Axiovert 40 CFL confocal microscope (Carl Zeiss, Oberkochen, Germany). Islet size was measured using iSolution DT 36 software (Carl Zeiss).

### Western blot and immunoprecipitation (IP)

Islets or cell lysates containing 20 μg were separated by 10% SDS-PAGE and transferred to PVDF membranes. After blocking with 5% skim milk, the blot was probed with primary antibody against Glut2, GK, TFAM, insulin (Santa Cruz Biotechnology), Pdx1 (Abcam, Cambridge, UK), Sirt6, FoxO1, p-FoxO1 (Cell Signaling Technology), and GAPDH (Bioworld Technology). For immunoprecipitation, 250 μg of protein precleared with protein G-agarose was incubated with anti-FoxO1 overnight at 4 °C, then with protein G-agarose at 4 °C for 2 h. Blots were probed with primary antibody against Sirt6, acetyl-lysine, FoxO1 (Cell Signaling Technology), or ubiquitin (Santa Cruz Biotechnology), and signals were detected with a Las-4000 imager (GE Healthcare Life Science, Pittsburgh, PA, USA).

### RNA isolation and real-time RT-PCR

Total RNA was extracted from islets using Trizol reagent (Invitrogen). RNA was precipitated with isopropanol and dissolved in diethylpyrocarbonate-treated distilled water. First-strand cDNA was generated using the random hexamer primer provided in the first-strand cDNA synthesis kit (Applied Biosystems, Foster City, CA). Specific primers for each gene (Supplementary Table 1) were designed using qPrimerDepot (http://mouseprimerdepot.nci.nih.gov). Real-time RT-PCR reactions comprised a final volume of 10 μl, containing 1 ng of reverse-transcribed total RNA, 2 nM of forward and reverse primers, and PCR master mixture. RT-PCR was performed in 384-well plates using an ABI Prism 7900HT Sequence Detection System (Applied Biosystems).

### Statistical analysis

Data are expressed as the mean ± SEM. Statistical comparisons were performed using one-way analysis of variance followed by Fisher’s post-hoc analysis. The significance of differences between groups was determined using Student’s unpaired *t*-test. A *p* value less than 0.05 was considered significant.

## Additional Information

**How to cite this article**: Song, M.-Y. *et al*. Insulin secretion impairment in Sirt6 knockout pancreatic β cells is mediated by suppression of the FoxO1-Pdx1-Glut2 pathway. *Sci. Rep.* **6**, 30321; doi: 10.1038/srep30321 (2016).

## Supplementary Material

Supplementary Information
