# Supplementary material for: Insulin secretion impairment in Sirt6 knockout pancreatic β cells is mediated by suppression of the FoxO1-Pdx1-Glut2 pathway
Source: Sci Rep. 2016 Jul 26;6:30321. doi: 10.1038/srep30321 (PMC4960548; doi:10.1038/srep30321)
Supplement: Supplementary Information [file srep30321-s1.pdf]

Insulin secretion impairment in Sirt6 knockout pancreatic β cells is mediated by suppression of the FoxO1-Pdx1-Glut2 pathway

Mi-Young Song[a], Jie Wang[a], Sun-O Ka[a], Eun Ju Bae[b,¶], and Byung-Hyun Park[a,¶]

[a]Department of Biochemistry, Chonbuk National University Medical School, Jeonju, Jeonbuk 54896, Republic of Korea

[b]College of Pharmacy, Woosuk University, Wanju, Jeonbuk 55338, Republic of Korea

Contents

1. Supplementary Table
2. Supplementary Figures Legends
3. Supplementary Figures

1. Supplementary Table

Table S1. Sequences and accession numbers for primers (forward, FOR; reverse, REV) used in real-time RT-PCR

Gene	Sequences for primers	Accession No.
Glut2	FOR: CCGAACTGGAAGGAACTCAG<br>REV: GGATTAAGCGGACAATTCCA	NM_031197
Pdx1	FOR: ACGGGTCCTCTTGTTTTCCT<br>REV: ACGGGTCCTCTTGTTTTCCT	NM_008814
GK	FOR: GGCTCATCACCTTCTTCAGG<br>REV: CTGGATGACAGAGCCAGGAT	NM_010292
FoxO1	FOR: TGCTGTGAAGGGACAGATTG<br>REV: GAGTGGATGGTGAAGAGCGT	NM_019739
Ins2	FOR: GGTCTGAAGGTCACCTGCTC<br>REV: GGAGCGTGGCTTCTTCTACA	NM_008387
FoxA2	FOR: TCATGTTGCTCACGGAAGAG<br>REV: TAAAGTATGCTGGGAGCCGT	NM_010446
NeuroD1	FOR: CGCTCTCGCTGTATGATTTG<br>REV: CGCTCTCGCTGTATGATTTG	NM_010894
NeuroD2	FOR: CTGGAGAGCTGCAGAACCG<br>REV: TTTTTCTTTGCCTTTGTCCG	NM_010895

2. Supplementary Figure Legends

Figure S1. Sirt6 expression in pancreatic islets. (A) Sirt6 protein levels were determined in mice islets treated with either cytokine mixture (1 U/ml of IL-1β and 100 U/ml of IFN-γ) or palmitate (1 mM) for 24 h or isolated from mice fed a normal chow diet (NCD) or high fat diet (HFD) for 16 weeks. **(B)** Histological analyses of pancreatic sections from multiple-low dose streptozotocin (MLDS)-treated mice, and *db/db* mice by H&E staining and immunostaining with antibodies against Sirt6, insulin, and glucagon. Bars=250 μm.

Figure S2. Generation of β cell-specific *Sirt6* KO mice. (A) Schematic illustration of *Rip2-Cre*-mediated deletion of exons 2 and 3 from the *Sirt6* allele. **(B)** Western blot analysis for Sirt6 in various tissues from WT and βS6KO mice. **(C)** Immunofluorescence staining for Sirt6 and insulin in pancreatic sections from WT and βS6KO mice.

Figure S3. Effects of β cell-specific Sirt6 deletion on glucose metabolism. All experimental conditions were the same as described in the Fig. 1 legend except for the use of 4-week old mice. Values are mean±SEM (n=8). $^{*}p<0.05$ and $^{**}p<0.01$ vs. WT.

Figure S4. Immunostaining of islets from WT and βS6KO mice. (A) Representative microphotographs of pancreatic sections from WT and βS6KO mice stained for insulin antibody. **(B)** Statistical analysis of the average size of the islets in pancreatic tissues (all islets in 10 whole sections from six WT mice and βS6KO mice were photographed and analyzed). **(C)** Immunofluorescence staining for insulin (green) and glucagon (red) in 12-week mice pancreas. **(D, E)** Pancreatic insulin contents and plasma glucagon levels were analyzed by specific ELISA. **(F)** Pancreatic tissues were TUNEL-stained, and the number of apoptotic islets was counted and expressed as a percentage of the total number of islets.

Values are mean±SEM (n=6).

Figure S5. Effects of Sirt6 on glucose-induced elevation of $[Ca^{2+}]_i$ and insulin secretion in MIN6 cells and isolated islets. (A) Following adenovirus infection, MIN6 cells were incubated with Fluo-4 AM for 40 min in KRB buffer before measuring $[Ca^{2+}]_i$ and then perfused with KRB buffer containing 2 or 20 mM glucose. $[Ca^{2+}]_i$ was calculated by the change in Fluo-4 fluorescence intensity. Stimulus (2 or 20 mM glucose) was presented at the time indicated by arrow. **(B)** A direct comparison of $[Ca^{2+}]_i$ level at 220 sec. **(C, D)** Basal and glucose-stimulated insulin secretion from MIN6 cells (C) and isolated islets from WT and βS6KO mice (D). Values are mean±SEM (n=6). $^{*}p<0.05$ and $^{**}p<0.01$ vs. AdLacZ or 20 mM glucose+Ad-LacZ; $^{\#}p<0.05$ and $^{\#\#}p<0.01$ vs. AdSirt6 or 20 mM glucose+WT.

Figure S6. Nuclear-to-cytosolic trafficking Sirt6 in MIN6 cells. All experimental procedures were the same as described in Fig. 4 legend except for the use of MIN6 cells. **(A)** Western blotting analysis of the subcellular localization of FoxO1. **(B)** Confocal microscopic images of the subcellular localization of FoxO1.

Figure S7. Regulation of FoxO1 protein stability by Sirt6. (A) MIN6 cells were co-transfected with either *Flag* or *Sirt6* along with WT *FoxO1* or 6KR mutant *FoxO1*. Forty-eight hours after transfection, cycloheximide (CHX, 80 μg/ml) was added to the culture media for the indicated times. Protein levels of FoxO1 and HSP90 were examined by Western blotting. **(B)** Densitometry was used to quantify the FoxO1 and HSP90 levels. The relative expression levels are the ratios of FoxO1 to HSP90 levels relative to those at 0 h. Values are mean±SEM (n=3). $^{**}p<0.01$ vs. Flag+WT; $^{\#\#}p<0.01$ vs. Flag+6KR.

Figure S8. Reduced mitochondrial biogenesis capacity and ATP content in βS6KO islets. (A) Islets were isolated from WT and βS6KO mice and protein level of TFAM was analyzed by Western blotting. **(B)** Islets isolated from c57BL6 mice were infected with control or Sirt6 adenoviruses, and then protein level of TFAM was compared. **(C)** ATP content in WT and βS6KO islets was quantified by the bioluminescent assay and normalized to the total protein content. Values are mean±SEM (n=8). $^{**}p<0.01$ vs. 2 mM glucose+WT; $^{\#\#}p<0.01$ vs. 20 mM glucose+WT.

3. Supplementary Figures

Figure S1

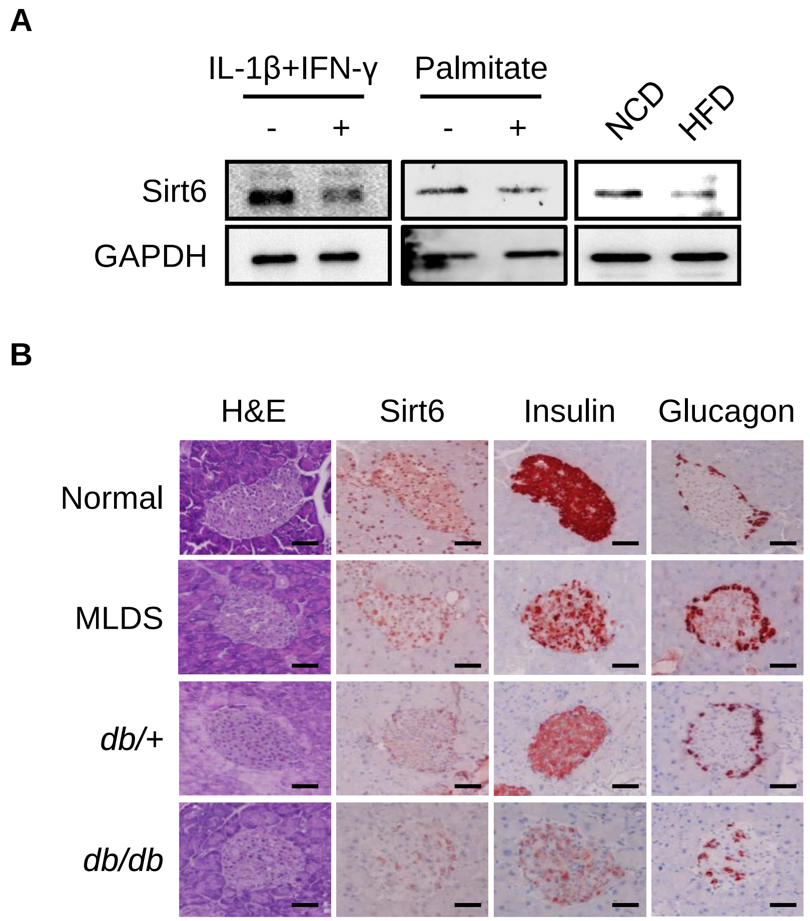
A
IL-1β+IFN-γ
Palmitate
-
+
-
+
NCD
HFD
Sirt6
GAPDH
B
H&E
Sirt6
Insulin
Glucagon
Normal
MLDS
db/+
db/db

Figure S2

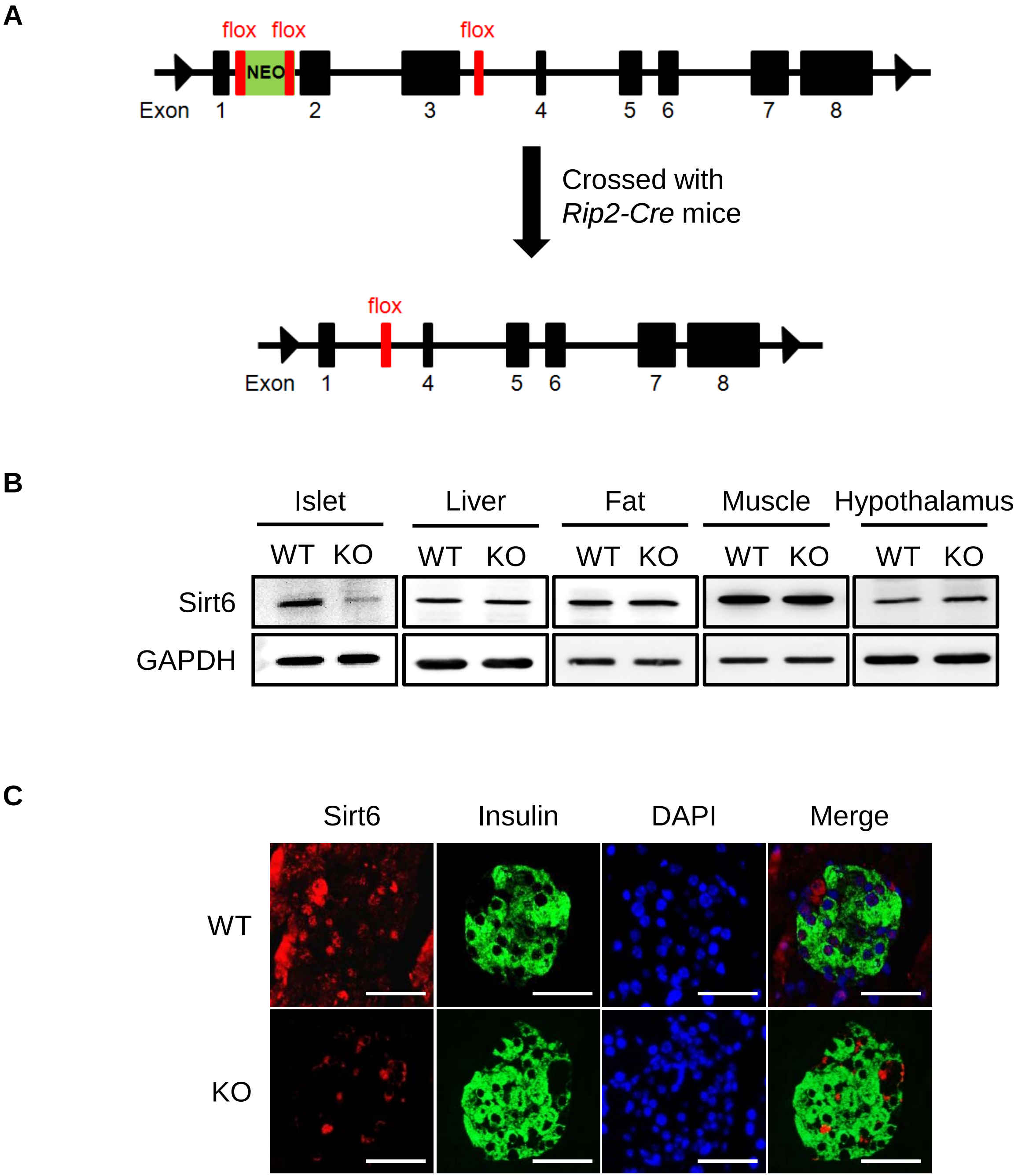
A
flox
flox
NEO
flox
Exon 1 2 3 4 5 6 7 8
Crossed with
Rip2-Cre mice
flox
Exon 1 4 5 6 7 8
B
Islet
Liver
Fat
Muscle
Hypothalamus
WT KO
Sirt6
GAPDH
C
Sirt6
Insulin
DAPI
Merge
WT
KO

Figure S3

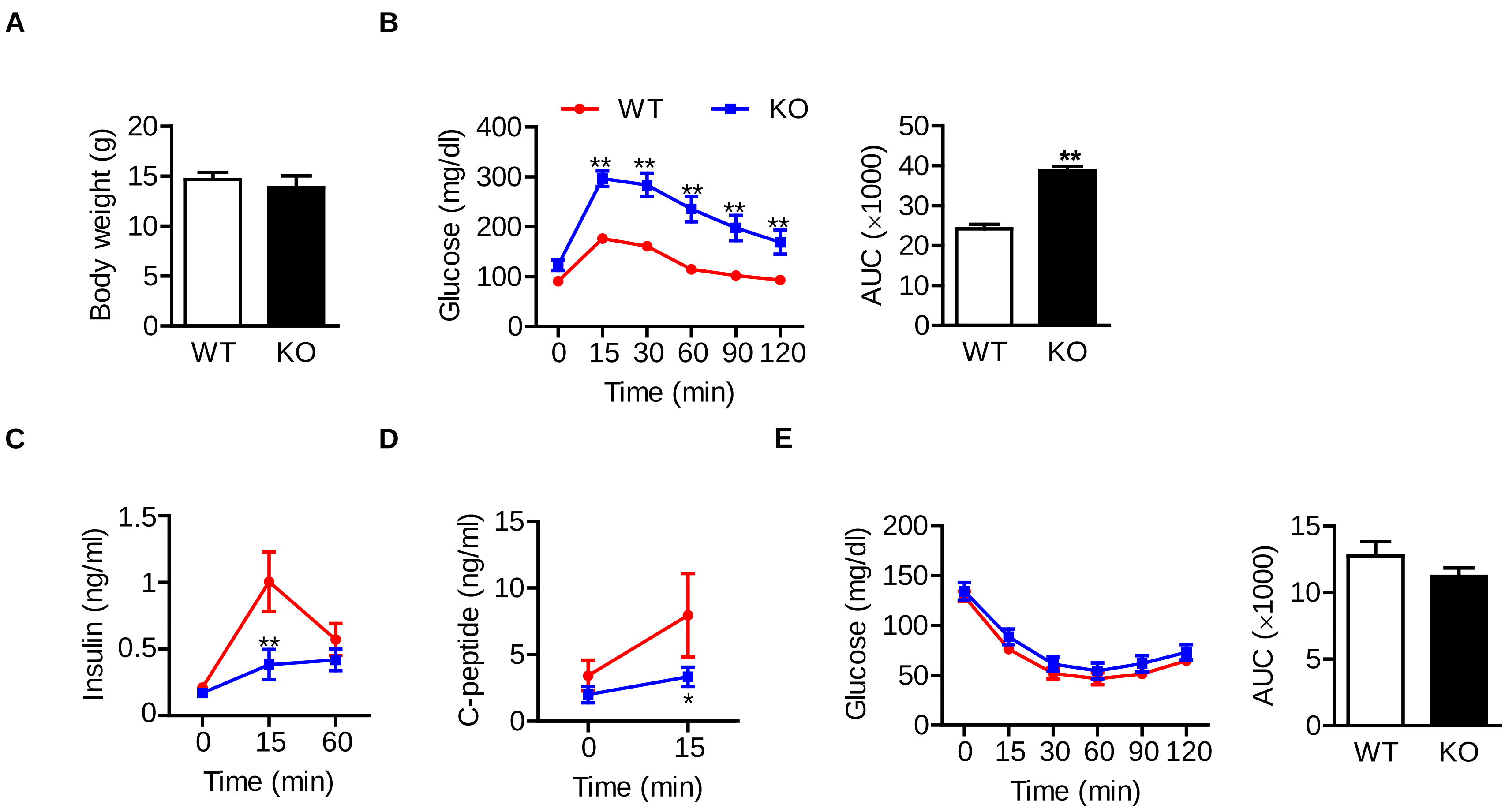
A
B
C
D
E
Body weight (g)
20
15
10
5
0
WT
KO
WT
KO
Glucose (mg/dl)
400
300
200
100
0
**
0
15
30
60
90
120
Time (min)
AUC (×1000)
50
40
30
20
10
Insulin (ng/ml)
1.5
1
0.5
C-peptide (ng/ml)
*
Glucose (mg/dl)
150
AUC (×1000)

Figure S4

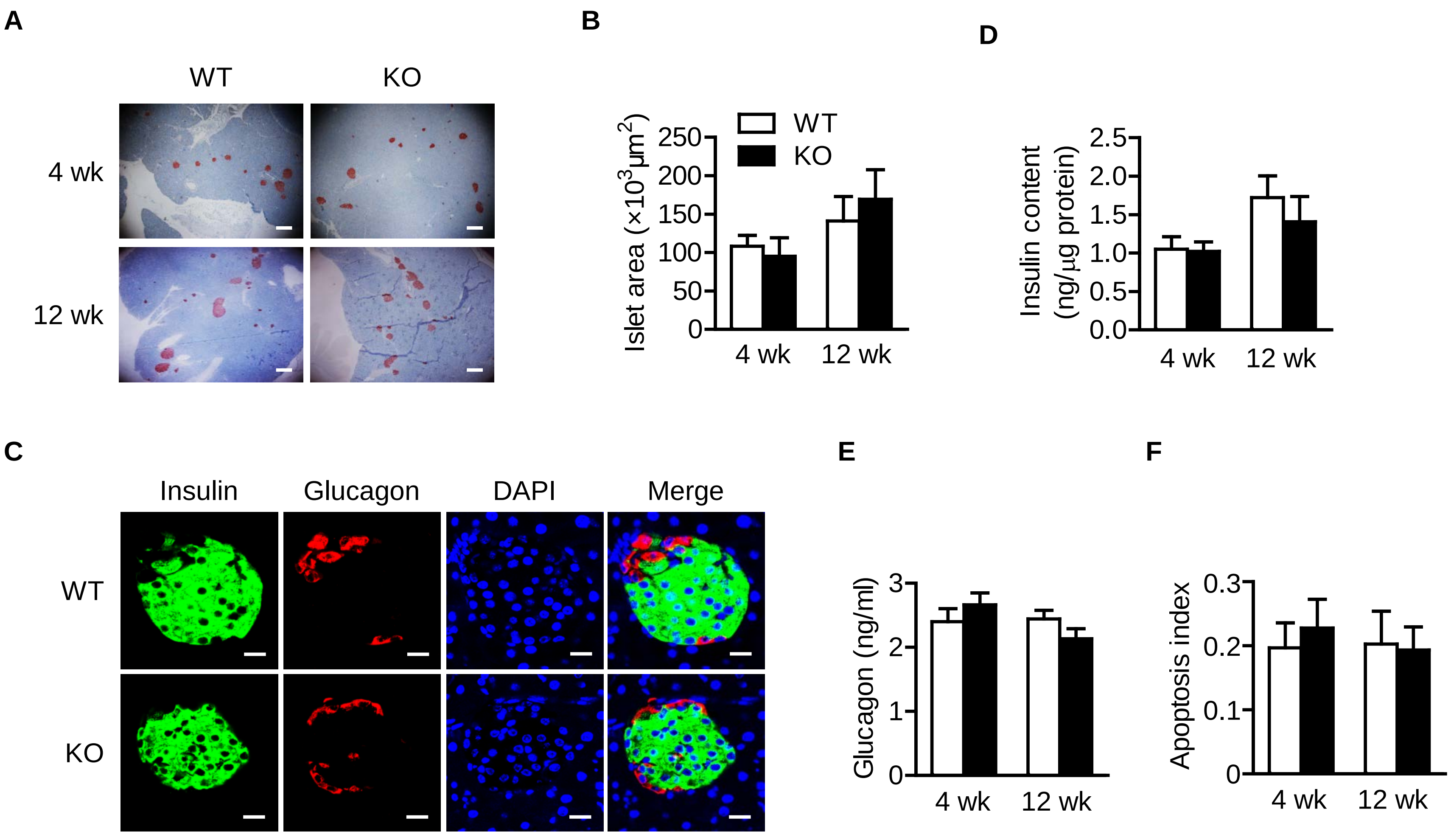
A
WT
KO
4 wk
12 wk
B
Islet area (×10³μm²)
250
200
150
100
50
0
WT
KO
4 wk
12 wk
D
Insulin content (ng/μg protein)
2.5
2.0
1.5
1.0
0.5
0.0
4 wk
12 wk
C
Insulin
Glucagon
DAPI
Merge
WT
KO
E
Glucagon (ng/ml)
3
2
1
0
4 wk
12 wk
F
Apoptosis index
0.3
0.2
0.1
0
4 wk
12 wk

Figure S5

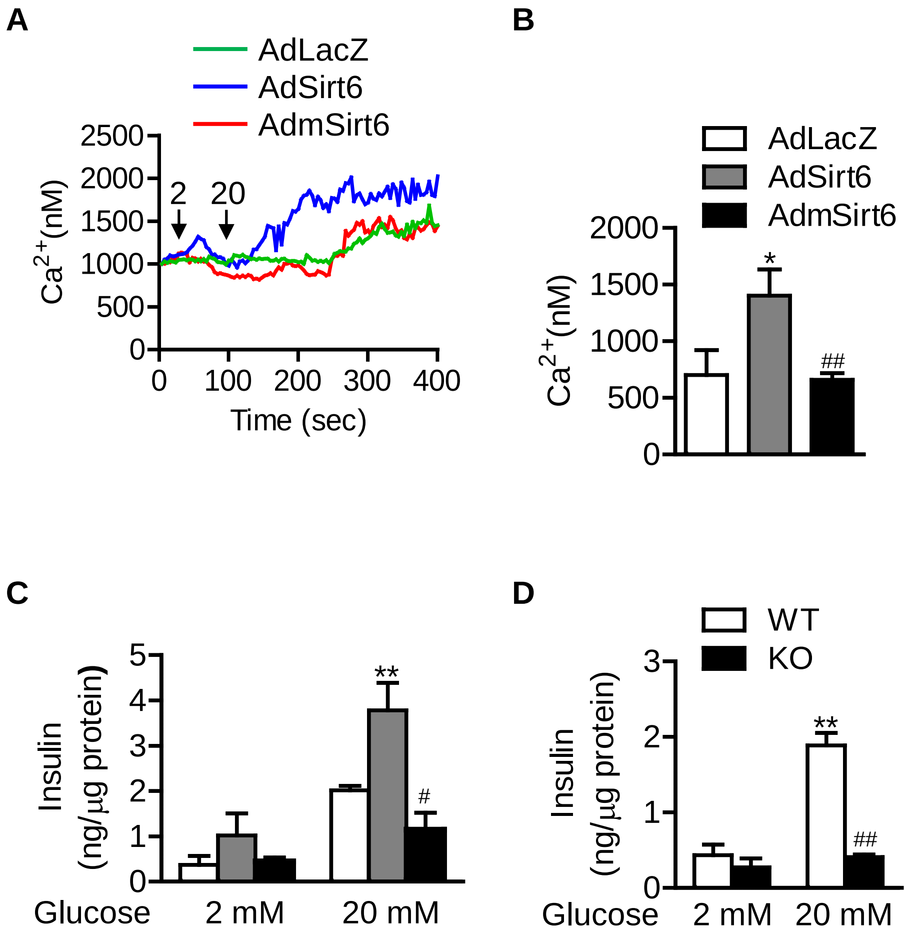
A
AdLacZ
AdSirt6
AdmSirt6
2500
2000
1500
1000
500
0
Ca2+(nM)
2
20
0
100
200
300
400
Time (sec)
B
AdLacZ
AdSirt6
AdmSirt6
2000
1500
1000
500
0
Ca2+(nM)
*
##
C
5
4
3
2
1
0
Insulin
(ng/μg protein)
**
#
Glucose
2 mM
20 mM
D
WT
KO
3
2
1
0
Insulin
(ng/μg protein)
**
##
Glucose
2 mM
20 mM

Figure S6

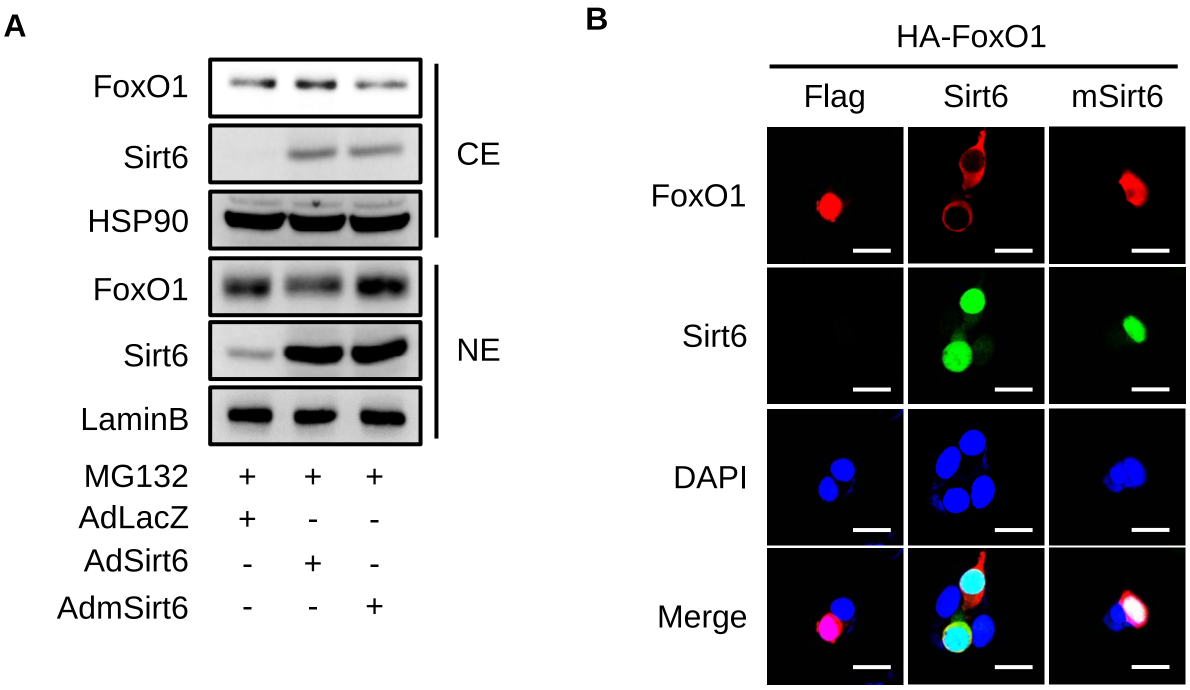
A
FoxO1
Sirt6
HSP90
CE
FoxO1
Sirt6
LaminB
NE
MG132 + + +
AdLacZ + - -
AdSirt6 - + -
AdmSirt6 - - +
B
HA-FoxO1
Flag
Sirt6
mSirt6
FoxO1
Sirt6
DAPI
Merge

Figure S7

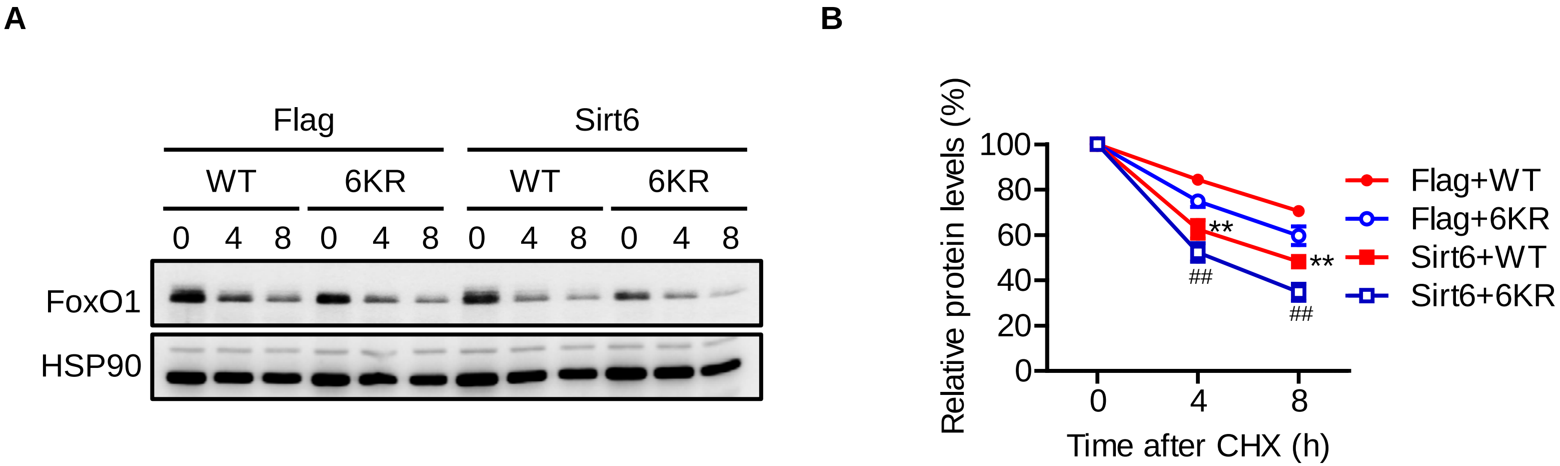
A
Flag
Sirt6
WT
6KR
WT
6KR
0 4 8 0 4 8 0 4 8 0 4 8
FoxO1
HSP90
B
Relative protein levels (%)
100
80
60
40
20
0
0
4
8
Time after CHX (h)
**
**
##
##
Flag+WT
Flag+6KR
Sirt6+WT
Sirt6+6KR

Figure S8

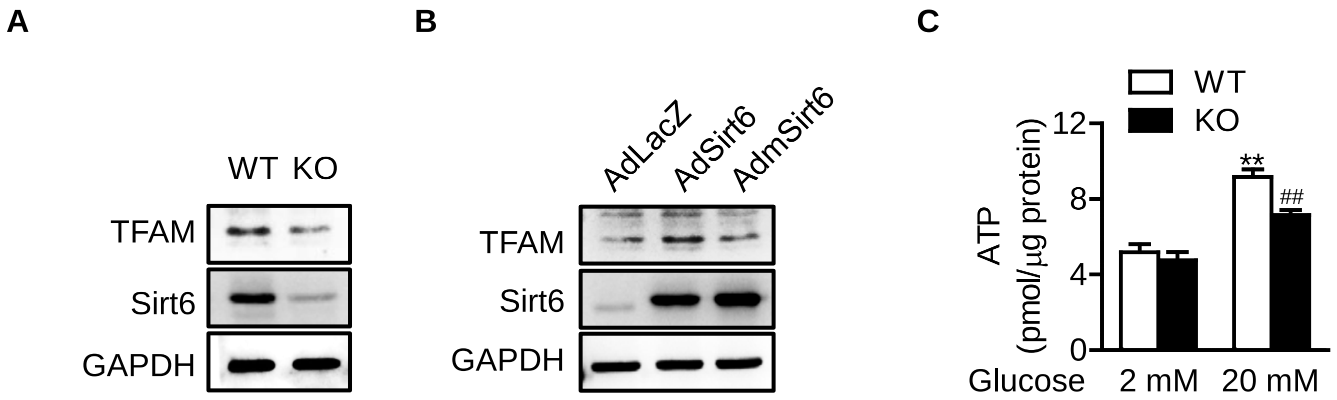
A
WT KO
TFAM
Sirt6
GAPDH
B
AdLacZ
AdSirt6
AdmSirt6
TFAM
Sirt6
GAPDH
C
WT
KO
ATP (pmol/μg protein)
12
8
4
0
**
##
Glucose
2 mM
20 mM
